# GABA and glutamate deficits from frontotemporal lobar degeneration are associated with disinhibition

**DOI:** 10.1093/brain/awaa305

**Published:** 2020-11-03

**Authors:** Alexander G Murley, Matthew A Rouse, P Simon Jones, Rong Ye, Frank H Hezemans, Claire O’Callaghan, Polytimi Frangou, Zoe Kourtzi, Catarina Rua, T Adrian Carpenter, Christopher T Rodgers, James B Rowe

**Affiliations:** a1 Department of Clinical Neurosciences, University of Cambridge, UK; a2 Cambridge University Hospitals NHS Foundation Trust, UK; a3 MRC Cognition and Brain Sciences Unit, University of Cambridge, UK; a4 Department of Psychiatry, University of Cambridge, UK; a5 Department of Psychology, University of Cambridge, UK; a6 Wolfson Brain Imaging Centre, University of Cambridge, UK

**Keywords:** frontotemporal dementia, progressive supranuclear palsy, GABA, glutamate, impulsivity

## Abstract

Behavioural disinhibition is a common feature of the syndromes associated with frontotemporal lobar degeneration (FTLD). It is associated with high morbidity and lacks proven symptomatic treatments. A potential therapeutic strategy is to correct the neurotransmitter deficits associated with FTLD, thereby improving behaviour. Reductions in the neurotransmitters glutamate and GABA correlate with impulsive behaviour in several neuropsychiatric diseases and there is post-mortem evidence of their deficit in FTLD. Here, we tested the hypothesis that prefrontal glutamate and GABA levels are reduced by FTLD *in vivo*, and that their deficit is associated with impaired response inhibition. Thirty-three participants with a syndrome associated with FTLD (15 patients with behavioural variant frontotemporal dementia and 18 with progressive supranuclear palsy, including both Richardson’s syndrome and progressive supranuclear palsy-frontal subtypes) and 20 healthy control subjects were included. Participants undertook ultra-high field (7 T) magnetic resonance spectroscopy and a stop-signal task of response inhibition. We measured glutamate and GABA levels using semi-LASER magnetic resonance spectroscopy in the right inferior frontal gyrus, because of its strong association with response inhibition, and in the primary visual cortex, as a control region. The stop-signal reaction time was calculated using an ex-Gaussian Bayesian model. Participants with frontotemporal dementia and progressive supranuclear palsy had impaired response inhibition, with longer stop-signal reaction times compared with controls. GABA concentration was reduced in patients versus controls in the right inferior frontal gyrus, but not the occipital lobe. There was no group-wise difference in partial volume corrected glutamate concentration between patients and controls. Both GABA and glutamate concentrations in the inferior frontal gyrus correlated inversely with stop-signal reaction time, indicating greater impulsivity in proportion to the loss of each neurotransmitter. We conclude that the glutamatergic and GABAergic deficits in the frontal lobe are potential targets for symptomatic drug treatment of frontotemporal dementia and progressive supranuclear palsy.

## Teaser

Murley *et al.* use ultra-high field (7T) magnetic resonance spectroscopy to measure *in vivo* glutamate and GABA in frontotemporal lobar degeneration syndromes, and show that deficits in these neurotransmitters are associated with behavioural disinhibition.

## Introduction

Behavioural change is a common feature of the syndromes associated with frontotemporal lobar degeneration (FTLD) pathology, including behavioural variant frontotemporal dementia (bvFTD) and progressive supranuclear palsy (PSP) (Gerstenecker *et al.*, 2013; Bang *et al.*, 2015; Lansdall *et al.*, 2017; Murley *et al.*, 2020*a*). This is associated with loss of functional independence (Agarwal *et al.*, 2019; Murley *et al.*, 2020*b*) and increased mortality (Lansdall *et al.*, 2019) in both disorders. Better treatment of behavioural symptoms might therefore improve both functionally independent survival and quality of life for patients and their families. A potential treatment strategy is to reverse neurotransmitter deficits, which has been effective in other neurodegenerative and neuropsychiatric disorders (Barone, 2010; Kandimalla and Reddy, 2017). There is evidence of neurotransmitter deficits in FTLD, but limited evidence of a relationship with phenotype (Huey *et al.*, 2006; Murley and Rowe, 2018).

The behavioural disturbance caused by FTLD syndromes comprises many neurocognitive processes with distinct anatomical and neurochemical alterations (Ranasinghe *et al.*, 2016; Passamonti *et al.*, 2018). An inability to inhibit inappropriate actions is seen in both bvFTD (O’Callaghan *et al.*, 2013*a*; Hughes *et al.*, 2018) and PSP (Gerstenecker *et al.*, 2013; Zhang *et al.*, 2016*a*). This phenotypic overlap between bvFTD and PSP is reflected in the MDS-2017 criteria for the PSP-F subtype (Höglinger *et al.*, 2017), along with frequent parkinsonism in bvFTD (Rowe, 2019). In this study, we therefore used a transdiagnostic approach to behavioural disinhibition (Husain, 2017), with ‘FTLD syndromes’ encompassing bvFTD, PSP-Richardson’s syndrome and PSP-Frontal syndrome. We measured glutamate and GABA concentrations *in vivo*, before testing the association of these neurotransmitter deficits with behavioural disinhibition.

The neurotransmitters glutamate and γ-aminobutyric acid (GABA) are associated with behavioural variability in health and neurological and psychiatric diseases. For example, GABA concentrations in CSF (Lee *et al.*, 2009) and prefrontal cortex (Boy *et al.*, 2011; Silveri *et al.*, 2013; Hermans *et al.*, 2018) inversely correlate with impulsivity and risky decision-making (Fujihara *et al.*, 2015). Proton magnetic resonance spectroscopy (^1^H-MRS) enables *in vivo* non-invasive measurement of glutamate and GABA, and has identified deficits in diseases associated with impulsivity (Ende, 2015; Yasen *et al.*, 2017). GABA deficits are seen in drug and alcohol addiction (Prisciandaro *et al.*, 2017; Li *et al.*, 2020), attention deficit hyperactivity disorder (Edden *et al.*, 2012; Ende *et al.*, 2016) and obsessive compulsive disorder (Zhang *et al.*, 2016*b*). There is also an association between glutamate, measured *in vivo* with MRS, and self-reported impulsivity in healthy adults (Schmaal *et al.*, 2012*a*; Coccaro *et al.*, 2013), personality disorders (Hoerst *et al.*, 2010), attention deficit hyperactivity disorder (Naaijen *et al.*, 2015; Ende *et al.*, 2016) and addiction (Schmaal *et al.*, 2012*b*). The direction of the relationship between glutamate, GABA and impulsive behaviour is complex and may depend on disease state (Ende, 2015), brain region (Dharmadhikari *et al.*, 2015; Naaijen *et al.*, 2015) and receptor subtype (Lee *et al.*, 2011; Hermans *et al.*, 2018).

There is preclinical and clinical evidence of GABA and glutamate deficits in FTLD (Murley and Rowe, 2018). For example, in transgenic tauopathy mouse models, there is impairment of both glutamatergic (Gascon *et al.*, 2014; Warmus *et al.*, 2014; Decker *et al.*, 2016) and GABAergic (Levenga *et al.*, 2014; Li *et al.*, 2017; Jiang *et al.*, 2018) neuron function. In post-mortem human studies of FTLD, glutamatergic pyramidal neurons (Ferrer, 1999; Henderson *et al.*, 2000) and receptors (Francis *et al.*, 1993; Procter *et al.*, 1999; Bowen *et al.*, 2008; Gascon *et al.*, 2014) are reduced. GABAergic neurons are markedly reduced in FTD (Ferrer, 1999) and PSP (Levy *et al.*, 1995), with loss of GABA_A_ receptors in some brain regions (Landwehrmeyer and Palacios, 1994; Suzuki *et al.*, 2002). Post-mortem GABA concentrations are decreased in the basal ganglia in bvFTD (Kanazawa *et al.*, 1988). There is also emerging evidence of *in vivo* glutamate deficits (Benussi *et al.*, 2019). MRS in bvFTD shows reduced glutamate/glutamine levels in the frontal and temporal lobes (Ernst *et al.*, 1997; Sarac *et al.*, 2008), and there is an inverse correlation between CSF glutamate levels and verbal agitation (Vermeiren *et al.*, 2013). PET studies have also shown loss of glutamate and GABA receptors (Foster *et al.*, 2000; Leuzy *et al.*, 2016).

In this study we use ultra-high field (7 T) ^1^H-MRS to measure glutamate and GABA *in vivo*. This method requires the target region (voxel) to be selected before each scan. We chose the right inferior frontal gyrus as our experimental region of interest. This region is critical for response inhibition (Aron *et al.*, 2004, 2014), as shown in structural (Aron *et al.*, 2003) and functional studies (Swann *et al.*, 2009; Levy and Wagner, 2011; Ye *et al.*, 2014; Rae *et al.*, 2015). In bvFTD, abnormal functional connectivity of the inferior frontal gyrus is associated with impulsivity (Hughes *et al.*, 2015, 2018). We also measured glutamate and GABA in a control region, the right occipital lobe, which is minimally affected by FTLD pathologies (Riedl *et al.*, 2014).

We tested two specific hypotheses: (i) GABA and glutamate levels are reduced in the frontal but not occipital cortex in subjects with bvFTD/PSP compared with controls, even after correction for atrophy; and (ii) the GABA and glutamate deficits in the frontal lobe of patients are associated with failure of response inhibition.

## Materials and methods

### Participant recruitment

Forty-four patients with bvFTD or PSP were recruited from the Cambridge Centre for Frontotemporal Dementia, the Cambridge Centre for Parkinson-Plus and the ‘Join Dementia Research’ patient register. All patients had a clinical assessment to confirm they met the diagnostic criteria for bvFTD (Rascovsky *et al.*, 2011), PSP-Richardson’s syndrome or PSP-Frontal syndrome (Höglinger *et al.*, 2017). Disease severity was assessed with the Clinical Dementia Rating scale modified for FTLD (Knopman *et al.*, 2008, 2011) and Progressive Supranuclear Palsy Rating Scale (Golbe and Ohman-Strickland, 2007). Twenty age- and sex-matched controls with no history of a neurological or psychiatric illness were recruited from the ‘Join Dementia Research’ database. Participants were asked to abstain from alcohol and PRN benzodiazepines or ‘Z-drugs’ for 24 h prior to the scan but continue their regular medications. No participants in the study were taking regular ‘Z-drugs’ or benzodiazepines. All participants gave written informed consent. The study had ethical approval from the Cambridge Central Research Ethics Committee (16/EE/0351; 16/EE/0084).

### Neuropsychology

Participants underwent cognitive and neuropsychological assessments including the Addenbrooke’s Cognitive Examination-Revised (ACE-R) (Mioshi *et al.*, 2006), Frontal Assessment Battery (FAB) (Royall, 2001), Hayling Sentence Completion test (Burgess and Shallice, 1997) and INECO Frontal Screening test (Torralva *et al.*, 2009). Each participant’s closest relative completed the Cambridge Behavioural Inventory-Revised (CBI-R) (Wear *et al.*, 2008) and Frontotemporal Dementia Rating Scale (FRS) (Mioshi *et al.*, 2010). We report the Hayling A + B score instead of total score (O’Callaghan *et al.*, 2013*b*; Martyr *et al.*, 2019).

### Stop-signal task

A stop-signal type response inhibition task was used to measure response inhibition (Ye *et al.*, 2014; Tsvetanov *et al.*, 2018) (Supplementary material and Supplementary Fig. 1). Participants were presented with a series of trials consisting of either go, no-go or stop trials and responded using a manual two-button box. On go trials, participants pressed the left button when shown a left-pointing black arrow and pressed the right button when shown a right-pointing black arrow. On stop trials, after a short and variable ‘stop-signal’ delay (SSD), the black arrow changed colour from black to red and a tone sounded at the same time (the stop signal). On stop trials, the SSD was varied using a staircase method to target a cumulative stop accuracy of 50% in each participant (see Tsvetanov *et al.*, 2018 for details). The starting SSD was calculated from 20 go trials at the start of each block. These trials were omitted from further analysis. On no-go trials, the SSD was set to zero. Participants were instructed to not respond if the arrow became red, suppressing their imminent response. Participants were given standardized instructions and asked to respond as quickly and accurately as possible. Participants were told neither to slow down on go trials, nor to wait for a possible stop signal (Verbruggen *et al.*, 2019). The task consisted of five blocks of 120 trials (go *n *=* *450, no-go *n *=* *51, stop *n *=* *99). Participants undertook a practice session of 20 trials prior to the first block.

We used the Dynamic Models of Choice toolbox in R (Version 3.6.1) to perform parametric Bayesian hierarchical analysis of the stop-signal task (Matzke *et al.*, 2013; Heathcote *et al.*, 2019). This method is described in detail elsewhere (Heathcote *et al.*, 2019). In brief, the model assumes a race between three independent processes: one corresponding to the stop process and two corresponding to go processes that match or mismatch the choice stimulus. A correct go response occurs when the matching go process finishes before the mismatching go process. Successful stop trials occur when the stop process finishes before either of the go processes. The model assumes that the finishing times of these processes follow an ex-Gaussian skewed distribution, which is typical for reaction time data (Heathcote *et al.*, 1991). We estimated the mean (μ), standard deviation (σ) and exponential decay (τ) of the ex-Gaussian distribution separately for each process. We included two attentional failure parameters that represent the probability that the go and stop processes fail to start (‘trigger failure’). We estimated these parameters hierarchically, so that parameters for individual participants were considered to be samples from corresponding group-level distributions. We fitted this hierarchical model separately for the patient and control groups. The Dynamic Models of Choice model has several advantages over other methods of calculating stop-signal reaction time (SSRT). First, it provides a distribution of plausible SSRT values, rather than a point estimate of SSRT, which may better reflect disease-related disinhibition (Matzke *et al.*, 2013). Second, the model accounts for attentional failures on go and stop trials, which occur when a participant fails to react to a go or stop signal. These ‘trigger failures’ are common in health (Matzke *et al.*, 2017) and diseases such as schizophrenia (Matzke *et al.*, 2017) and, if not modelled, may cause overestimation of the SSRT (Matzke *et al.*, 2019; Skippen *et al.*, 2019). Third, the model can accommodate choice errors by including two Go runners, which yields more accurate parameter estimates for the stop process (Heathcote *et al.*, 2019; Matzke *et al.*, 2019). Lastly, hierarchical Bayesian methods regularize participant-level estimates according to group statistics, which enables reliable group-level inference and produces, on average, more accurate participant-level estimates (Gelman *et al.*, 2013).

We used Markov chain Monte Carlo sampling to approximate the posterior distributions of parameters simultaneously at the level of the group and individual participants. The prior distributions for the group-level parameters were the same as used by the model developers (Heathcote *et al.*, 2019), except for slightly higher prior mean values for μ_go-match_ (1.5 s), μ_go-mismatch_ (1.5 s) and μ_stop_ (1 s), to account for slower reaction times in older age and neurodegenerative disease. We initially ran the model using 33 chains (i.e. three times the number of parameters), with thinning of every 10th sample and a 5% probability of migration for both the group and participant levels. We assessed convergence of the Markov chain Monte Carlo chains by visual inspection of the trace plots and confirmed that the potential scale reduction statistic $\hat{R}$ was <1.1 for all parameters. After this, we obtained an additional 500 iterations for each chain to create a final posterior distribution of each parameter, for further analyses. We compared the observed and simulated data (generated from the model’s posterior predictive distribution), to ensure that the model adequately captures the data-generating process. The primary outcome of interest, SSRT, now without the potential confound of attentional failure, was computed as the sum of μ_stop_ and τ_stop_ (Matzke *et al.*, 2019).

### Magnetic resonance spectroscopy

Participants underwent scanning with a MAGNETOM Terra scanner (Siemens Healthineers) with a 32-channel receiver and single channel transmit head coil (Nova Medical). A T_1_-weighted MP2RAGE structural sequence [repetition time = 4300 ms, echo time = 1.99 ms, resolution = 99 ms, bandwidth = 250 Hz/px, voxel size = 0.75 mm^3^, field of view = 240 × 240 × 157 mm, acceleration factor (A ≫ P) = 3, flip-angle = 5/6° and inversion times = 840/2370 ms] was acquired for voxel placement and partial volume correction. The default settings for tissue probability parameters (six tissue classes) in the standard SPM12 pipeline were used for tissue segmentation and voxel-based morphometry (Supplementary material).

**Figure 1:**
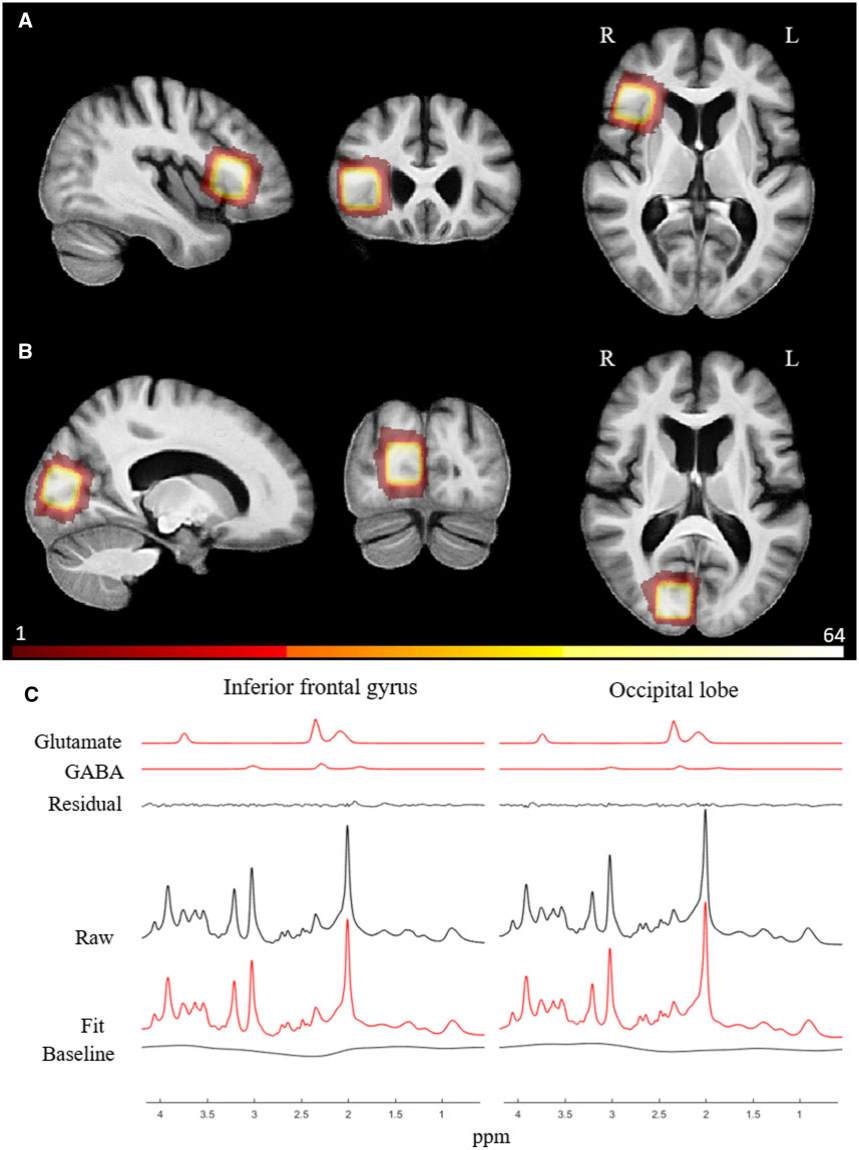
**Spectroscopy voxel location and composition.** (**A**) Frontal voxel (sum of all participants) superimposed on a mean structural image of all participants. (**B**) Occipital voxel location. (**C**) Mean spectra from all participants showing the raw data, LCModel fit, baseline, residual (fit-raw data), glutamate and GABA fits.

Magnetic resonance spectra were acquired serially from one region of interest, the right inferior frontal gyrus, and one control region, the right primary visual cortex. Voxel order was randomized between participants. Both voxels (2 × 2 × 2 cm^3^) were placed manually by the same operator (A.G.M.) using anatomical landmarks. To confirm that spectroscopy voxel placement was consistent across participants in both brain regions, we retrospectively overlaid the co-registered voxels on a T_1_ study-wise template. (Fig. 1A and B). Spectra were acquired using a short-echo semi-LASER sequence (Öz and Tkáč, 2011; Deelchand *et al.*, 2015) (repetition time/echo time = 5000/26 ms, 64 repetitions) using the recommended pre-scan protocol of FASTESTMAP shimming (Gruetter and Tkáč, 2000) semi-LASER water-peak flip angle and VAPOR water suppression calibration (Tkac *et al.*, 1999). This spectroscopy sequence gives reliable and reproducible GABA and glutamate measurements in the human brain *in vivo* (Barron *et al.*, 2016; Kolasinski *et al.*, 2017; Joers *et al.*, 2018; Frangou *et al.*, 2019; Hong *et al.*, 2019; Ip *et al.*, 2019).

Each of the 64 individual spectral transients from each participant were saved separately. These were then corrected for effects of eddy currents and for frequency and phase shifts using MRspa (Dinesh Deelchand, University of Minnesota, www.cmrr.umn.edu/downloads/mrspa). Two patient participants had inadequate data for further analysis and were excluded, due to incomplete scans and movement artefacts.

Neurochemicals between 0.5 and 4.2 ppm, including glutamate and GABA, were quantified using LCModel (Version 6.2-3) (Provencher, 1993), with water scaling and a simulated basis set that included experimentally-acquired macromolecule spectra (Fig. 1C). For partial volume correction, fractions of grey matter, white matter and CSF were obtained from segmentation of the MP2RAGE images using SPM12. A generalized linear model was used to remove the effect of age, sex and partial volume and the residual glutamate and GABA values were used for further analysis (Supplementary material).

### Statistical analysis

Analysis of variance was used to compare GABA and glutamate levels between groups, with region of interest as a within subject factor and diagnosis as a between subject factor. All *P*-values were corrected for multiple comparisons using Tukey’s test. We tested the association between the right inferior frontal gyrus GABA and glutamate concentrations and behavioural disinhibition, as measured by the SSRT and carer questionnaires, in participants with bvFTD/PSP. For each value in the individual-level posterior distributions of SSRT, a Spearman’s correlation coefficient was calculated with the residual glutamate and GABA values after partial volume, age and sex correction. This results in a posterior distribution of plausible correlation values (Ly *et al.*, 2018). The region of practical equivalence was defined as a Spearman’s R between −0.1 and 0.1, corresponding to a small effect size (Cohen, 1992; Kruschke, 2018). The null hypotheses was rejected if the 95% highest density interval (HDI) of the ‘R’ correlation values did not overlap with the region of practical equivalence (Kruschke, 2018). Analysis was performed in MATLAB 2018b (MathWorks, USA) and JASP (Version 0.11).

### Data availability

Anonymized data are available on reasonable request for academic purposes.

## Results

Forty-four patients with bvFTD/PSP participated in the study. The primary clinical diagnoses were evenly split between bvFTD (*n *=* *22) and PSP (*n *=* *22), but if MAX-rules and mutual exclusivity criteria were set aside (Grimm *et al.*, 2019), many patients met more than one set of diagnostic criteria for bvFTD, PSP-Frontal syndrome and PSP-Richardson’s syndrome. Thirty-six patients met the diagnostic criteria for probable bvFTD (with or without parkinsonism and oculomotor deficits), 19 met the criteria for PSP-Frontal syndrome and 23 met the criteria for PSP-Richardson’s syndrome (with or without cognitive and behavioural change). Fifteen patients exhibited clinical and radiological features consistent with all three conditions. Three patients with bvFTD had parkinsonism but did not meet the diagnostic criteria for PSP. Therefore, we use a transdiagnostic approach when reporting these results and refer to all patients with bvFTD or PSP as an ‘FTLD’ group, noting the high, but not perfect, clinical pathological correlations between clinically probable and possible bvFTD, PSP and the pathologies of FTLD (Perry *et al.*, 2017; Gazzina *et al.*, 2019).

Patient demographics and neuropsychology results are shown in Table 1. Statistical comparisons of the FTLD subgroups (bvFTD versus PSP) are included in the Supplementary material, noting that both groups were impulsive, as expected.

**Table 1:** Demographics and neuropsychology results of the study cohort

	Control mean (SD)	bvFTD/PSP mean (SD)	*t*-statistic	*P*-value
*n*	20	44		
Age, years	67.1 (5.6)	66.2 (8.4)	0.47	NS
Sex, %male	65	72	0.263^a^	NS
Disease onset to study, years (SD)	NA	5.84 (11.32)	NA	NA
Diagnosis to study, years (SD)	NA	1.08 (1.48)	NA	NA
CDR^®^ plus NACC FTLD	0 (0)	9.81 (5.19)	–8.42	<0.001
PSPRS Total	0.1 (0.31)	23.1 (17.81)	–5.75	<0.001
ACER Total	96.2 (2.71)	68 (24.11)	5.20	<0.001
FAB	17.45 (0.83)	11.14 (5.14)	5.62	<0.001
Hayling (A+B score)	4.3 (7.12)	24.31 (19.58)	–4.08	<0.001
Hayling Total	18.45 (2.28)	11.26 (4.83)	5.86	<0.001
INECO	25.78 (2.83)	14.2 (7.53)	6.63	<0.001
CBIR Total	6.35 (6.13)	64.62 (36.52)	–6.61	<0.001
FRS Total (Logit)	0.86 (0.3)	0.37 (0.28)	9.23	<0.001

ACER = Addenbrooke’s Cognitive Examination-Revised; CBIR = Cambridge Behavioural Inventory-Revised; CDR® plus NACC FTLD = Clinical Dementia Rating Scale plus NACC FTLD behaviour and language domains, sum of boxes; FAB = Frontal Assessment Battery; FRS = Frontotemporal Dementia Rating Scale; NA = not applicable; PSPRS = Progressive Supranuclear Palsy Rating Scale; SD = standard deviation.

^a^ Chi-squared, NS (not significant) = *P *>* *0.05

First, we compared grey and white matter volumes between FTLD syndromes and healthy controls using voxel-based morphometry. Participants with FTLD had reduced grey matter volume in the frontal and temporal lobes, basal ganglia, thalamus and cerebellum, with corresponding white matter volume loss in the frontostriatal and corticospinal tracts and brainstem. Brain volume was relatively preserved in the occipital lobe (Supplementary Fig. 2). Participants with bvFTD and those with PSP had reduced grey matter in the right orbitofrontal and anterior cingulate cortex, bilateral inferior frontal gyri, insula and motor cortices, as shown by a conjunction analysis (Nichols *et al.*, 2005). This also revealed volume loss in subcortical structures including the caudate, putamen and globus pallidus and superior cerebellum. White matter volume loss was seen in frontostriatal pathways (Supplementary Fig. 3).

**Figure 2:**
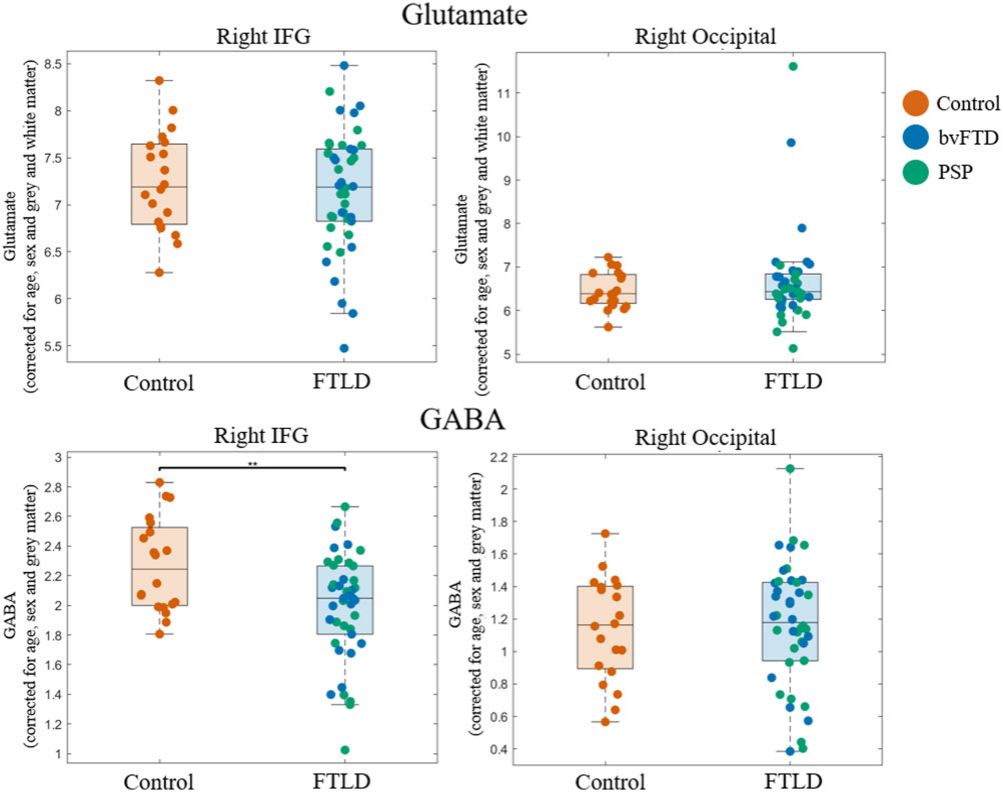
**Glutamate and GABA concentrations in the FTLD syndromes of bvFTD and PSP.** Values are corrected for age, sex and partial volume (grey and white matter for glutamate, grey matter for GABA). **P =* *0.005. IFG = inferior frontal gyrus.

Second, we used ^1^H-MRS to measure glutamate and GABA concentrations in the right inferior frontal gyrus and occipital lobe. The spectral quality was adequate for neurotransmitter quantification in both brain regions (Table 2 and Fig. 1C). The mean correlation coefficients between all metabolites and both GABA and glutamate were less negative than −0.3, suggesting both were accurately distinguished from other metabolites (Provencher, 1993). GABA and glutamate measurements were water scaled, then corrected for partial volume, age, sex and measurement accuracy (Supplementary material). Water-scaled values without correction are shown in the Supplementary material. There was no difference between groups in glutamate concentration in either voxel [simple main effects: right inferior frontal gyrus *F*(1) = 0.34, *P *=* *0.56; right occipital lobe *F*(1) = 0.73, *P *=* *0.40] (Fig. 2). GABA was reduced in bvFTD/PSP compared to controls in the right inferior frontal gyrus [*F*(1) = 8.67, *P *=* *0.005] but not occipital lobe [*F*(1) = 0.06, *P *=* *0.81] (Fig. 2). Including white matter volume in the regression analysis for GABA concentrations did not change this finding. The GABA deficit in the right inferior frontal gyrus was present in both bvFTD [*t*(38) = 2.93, *P *=* *0.006] and PSP [*t*(40) = 2.36 *P *=* *0.023] subgroups compared with the healthy controls. Removing the one outlier (Grubb’s test *P *<* *0.05) in the occipital lobe region in the patient group did not change these results.

**Table 2:** Spectral quality measurements

	Control mean (SD)	bvFTD/PSP mean (SD)	*t*-statistic	*P*-value
Water line width, Hz				
Right inferior frontal gyrus	13.9 (1.3)	13.0 (2.7)	1.86	0.18
Right occipital lobe	13.9 (1.0)	13.34 (1.7)	2.11	0.15
Signal-to-noise ratio				
Right inferior frontal gyrus	54.8 (5.3)	44.8 (8.7)	22.25	<0.001
Right occipital lobe	67.4 (15.38)	57.8 (14.1)	5.39	0.02
Glutamate CRLB				
Right inferior frontal gyrus	2.1 (0.3)	2.4 (0.5)	4.7	0.034
Right occipital lobe	2.3 (0.7)	2.4 (1.1)	0.22	0.65
GABA CRLB				
Right inferior frontal gyrus	9.4 (1.1)	12.3 (4.4)	8.21	0.006
Right occipital lobe	19.2 (17.3^a^)	19.4 (9.3)	0.005	0.946

Values are presented as mean and standard deviation for each group. CRLB = Cramér Rao Lower Bound.

^a^ There was one outlier in the control group (CRLB 84).

Third, we used Bayesian hierarchical modelling of a stop-signal task to estimate the SSRT as the measure of response inhibition. Data from nine participants with bvFTD/PSP were excluded, due to low number of trials (<50 stop trials) or inability to complete the task. These excluded participants did not have significantly different neurotransmitter concentrations from the group that completed the task [right inferior frontal gyrus GABA *t*(40) = 0.47, *P *=* *0.64; glutamate *t*(40) = 0.56, *P *=* *0.58]. The remaining bvFTD/PSP (bvFTD *n *=* *17, PSP *n *=* *18) and control participants completed a similar total number of trials (mean 663 versus 670 trials, Mann-Whitney U-test = 300, *P *=* *0.228) but participants with bvFTD/PSP made more go errors (Mann-Whitney U-test = 185.5, *P *=* *0.003) and omissions (Mann-Whitney U-test = 231.5, *P *=* *0.005). Further details regarding behavioural performance are provided in the Supplementary material.

**Figure 3:**
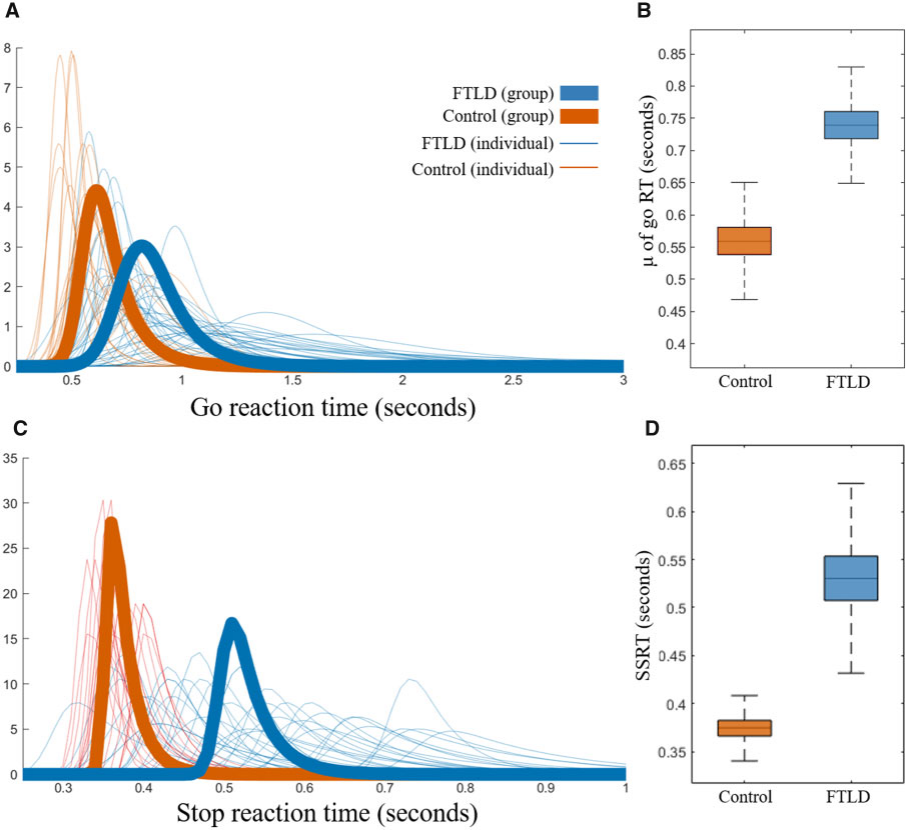
**Reaction time distributions for patients with bvFTD/PSP and healthy controls.** The group-level result is shown by the thick line and individual-level results are shown by the thinner lines. (**A**) Go reaction time distributions. (**B**) Box plot of 95% highest density interval for the µ of the go reaction time. bvFTD/PSP = blue; healthy controls = red.

The stop-signal task performance descriptive results, Markov chain Monte Carlo trace plots and prior and posterior density plots are shown in the Supplementary material. The posterior estimates for the group and individual level go and stop reaction time distributions for bvFTD/PSP syndromes and controls are shown in Fig. 3. All control individual-level reaction times were similar to the group-level distribution, with no evidence of strategic slowing. In bvFTD/PSP, individual go reaction time distributions varied widely; some overlapped with the control distributions, but many were markedly longer (Fig. 3A). There was also similar variability in bvFTD/PSP stop reaction time distributions (Fig. 3C).

There was a group-level difference in SSRT between the participants with bvFTD/PSP and controls, with clear separation of the ex-Gaussian distributions and no overlap in the 95% HDIs of the mean reaction time (Fig. 3D). The go reaction time did not differ significantly between groups, as evidenced by the overlapping HDI boundaries (Fig. 3B).

**Figure 4:**
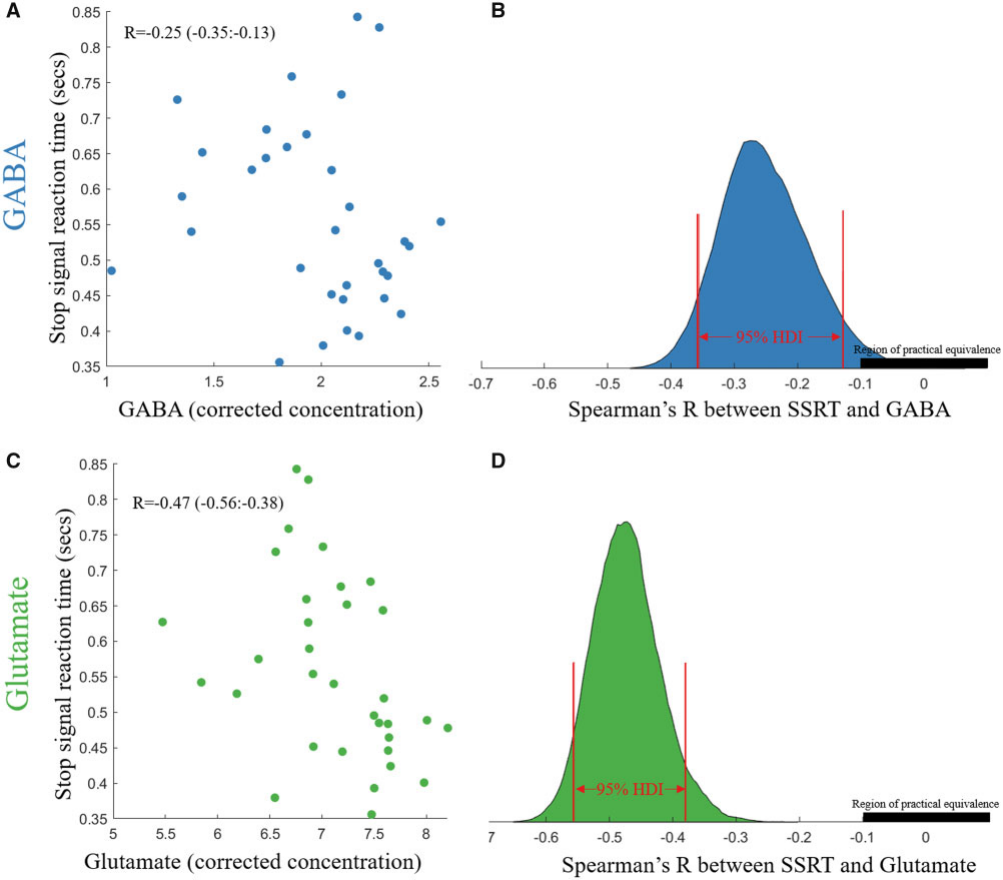
**Correlation between neurotransmitters (GABA and glutamate) and SSRT.** Results from bvFTD/PSP patients only. (**A**) Scatter plot of mean SSRT and corrected GABA, values in brackets are 95% HDI. (**B**) Histogram of Spearman’s correlation values between glutamate (corrected for grey matter, age and sex) and SSRT. Red lines show 95% HDI. Black bar shows region of practical equivalence (−0.1, 0.1). (**C**) Histogram of Spearman’s correlation values between glutamate and SSRT. (**D**) Scatter plot of mean SSRT and corrected glutamate.

Next, we tested the hypothesis that GABA and glutamate deficits in the right inferior frontal gyrus are associated with impulsivity in patients who underwent MRS and completed the stop signal task (bvFTD *n *=* *15, PSP *n *=* *18). Both GABA and glutamate concentrations in the right inferior frontal gyrus were inversely correlated with the SSRT (Fig. 4). This association with impaired response inhibition was stronger for glutamate (95% HDI: −0.56, −0.38) than GABA (95% HDI: −0.35, −0.13), but both these credible intervals were outside the prespecified region of practical equivalence (−0.1, 0.1). The corrected glutamate and GABA concentrations did not correlate (Spearman’s R = 0.06, *P *=* *0.70).

Finally, we tested the specificity of the association between GABA and glutamate concentrations in the right inferior frontal gyrus and SSRT. Trigger failure probability, a measure of inattention derived by the Dynamic Models of Choice model, was not associated with either the GABA (95% HDI: −0.13, 0.18) or glutamate (95% HDI: −0.01, 0.31) concentration in the inferior frontal gyrus (Supplementary Fig. 9). There was no association between SSRT and other MRS-visible metabolites, including *N*-acetylaspartate (95% HDI −0.25, −0.05), creatine/phosphocreatine (95% HDI −0.03, −0.19), glycerophosphocholine/phosphocholine (95% HDI −0.01, 0.23), myo-inositol (95% HDI −0.25, −0.03), glutamine (95% HDI −0.01, 0.11) and glutathione (95% HDI −0.10, 0.11). Occipital lobe GABA and glutamate concentrations were not associated with SSRT in either group. There was no association between neurotransmitter concentrations in any region and carer ratings of challenging behaviour (Supplementary material). There was no association between neurotransmitter concentration and SSRT or trigger failure probability in healthy volunteers.

## Discussion

This study has two main findings. First, GABA and glutamate levels are reduced in the right inferior frontal gyrus in patients with bvFTD and PSP, with the GABA deficit persisting after correction for age, sex and atrophy. Second, glutamate and GABA concentrations in the inferior frontal gyrus correlate with disinhibition, as measured by the SSRT.

This finding of a frontal lobe GABA deficit, as measured using ^1^H-MRS, is supported by other *in vivo* and post-mortem evidence of GABAergic neuron loss in bvFTD and PSP (Ferrer, 1999; Levenga *et al.*, 2014). A GABAergic deficit may contribute to the abnormal functional connectivity associated with cognitive impairment in FTLD syndromes. GABAergic interneurons have widespread functions beyond simple inhibition of excitatory neurons and have a key role in the regulation of oscillatory dynamics (Owens and Kriegstein, 2002; Mann and Paulsen, 2007; Buzsáki and Wang, 2012). Gamma and beta oscillation frequency correlates with cortical GABA concentration (Muthukumaraswamy *et al.*, 2009; Gaetz *et al.*, 2011; Kujala *et al.*, 2015; Baumgarten *et al.*, 2016) and GABA_A_ receptor density (Kujala *et al.*, 2015), while inhibition of GABAergic receptors reduces oscillatory power and impairs inhibition and working memory (Hines *et al.*, 2013). Beta-power correlates with behavioural disturbance in bvFTD (Hughes *et al.*, 2018), and bvFTD reduces frontotemporal beta-coherence (Hughes and Rowe, 2013). Brain network connectivity of the inferior frontal gyrus is altered in FTLD, during response inhibition (Hughes *et al.*, 2015, 2018) and at rest (Seeley *et al.*, 2009; Sami *et al.*, 2018). These altered oscillations and frequency-bound connectivities in bvFTD may be caused partially by GABAergic deficits. This raises the possibility that correcting GABAergic deficits may restore neurophysiological function and improve cognition and behaviour.

There was no difference in glutamate concentration between patients with bvFTD/PSP and controls after correction for grey and white matter volume loss. However, it would be misleading to conclude that there is no glutamatergic abnormality in FTLD syndromes. Given the high density of glutamatergic neurons in the neocortex, grey matter atrophy typically correlates with the number of glutamatergic neurons in the remaining brain tissue (Harding, 1998; Zarow *et al.*, 2005). Unlike GABA, glutamate has many functions in the CNS beyond neurotransmission including neuron and glia metabolism and protein synthesis (Hertz, 2013; Zhou and Danbolt, 2014). Only a small proportion of total glutamate acts as a neurotransmitter (Danbolt, 2001). Therefore, it is possible that MRS of glutamate is an indirect measure of glutamatergic neuron density. Correcting MRS measures of glutamate for atrophy would, in this case, have removed a difference between the results obtained from the participants with bvFTD/PSP and controls.

In the right inferior frontal gyrus voxel, both GABA and glutamate concentrations inversely correlated with disinhibited behaviour (impaired response inhibition, as measured by the SSRT). This complements results obtained with other functional imaging modalities, including functional MRI and electrophysiology, that show activation of the right inferior gyrus during the stop-signal task in healthy volunteers (Chambers *et al.*, 2006, 2009; Levy and Wagner, 2011; Aron *et al.*, 2014; Ye *et al.*, 2014; Rae *et al.*, 2015). The right inferior gyrus forms part of a cognitive control network, which is activated during response inhibition and also includes the presupplementary motor area and subthalamic nucleus (Rae *et al.*, 2015). GABA levels in this network, specifically the presupplementary motor area, inversely correlate with SSRT in healthy older adults (Hermans *et al.*, 2018), although Hermans *et al.* used an edited MRS sequence at 3 T and did not measure glutamate levels. One strength of our 7 T MRS study is that both glutamate and GABA can be measured at the same time in the same brain region to study whether both contribute to response inhibition in FTLD syndromes.

There was no association between GABA and glutamate concentrations in the right inferior gyrus and carer ratings of global behavioural impairment. This might be because the right inferior gyrus is just one of many regions associated with the socially disinhibited and challenging behaviours reported by carers. It cannot be assumed that GABA and glutamate concentrations in the right inferior gyrus are representative of the whole frontal lobe. Global behavioural impairment results from pathology in multiple brain regions and impairment in diverse cognitive processes. New sequences measuring glutamate and GABA across the whole brain may show correlation with other behavioural impairments in FTLD syndromes and are a promising area for future research (Moser *et al.*, 2019). In addition, deficits in other neurotransmitter pathways, including serotonin, dopamine, noradrenaline and acetylcholine also contribute to behavioural impairment in FTLD syndromes (Huey *et al.*, 2006; Hughes *et al.*, 2015; Murley and Rowe, 2018). Ultimately, an effective treatment for behavioural symptoms in FTLD may need to restore multiple neurotransmitter pathways.

This study has several limitations. First, MRS measurement accuracy is limited by scan quality (Wilson *et al.*, 2019). To mitigate this, we used a validated sequence, with automated shimming and water and outer volume suppression, that is recommended by recent consensus guidelines (Öz *et al.*, 2020). Our measures of MRS quality, including water linewidth, signal-to-noise ratio and Cramér-Rao lower bounds, were within standard limits for ultra-high field ^1^H-MRS (Wilson *et al.*, 2019; Öz *et al.*, 2020). In addition, the absence of a group difference in the control region (occipital lobe) suggests the results in the inferior frontal gyrus reflect a true neurotransmitter deficit and not an artefact of movement or another patient-related bias. Second, the spectroscopy regions of interest may have varied between individuals, particularly in the proportion of brain included, because participants had different total brain volumes, but their MRS voxels remained the same size. This was necessary to avoid a confound of varying signal-to-noise but means that the region of interest covers a slightly different proportion of the brain between participants. Third, brain volume within the MRS voxel was lower in the groups of patients with bvFTD/PSP. The GABA and glutamate concentrations of CSF are not high enough to be MRS-visible; therefore, this partial volume effect must be considered when reporting MRS results (Quadrelli *et al.*, 2016; Porges *et al.*, 2017). One approach is to report the relative concentration of the metabolite of interest to an internal standard, using another metabolite such as creatine. However, this was not appropriate in our patient group, where the creatine level is likely also to be abnormal, because of impaired metabolism (Foster *et al.*, 1988; Diehl-Schmid *et al.*, 2007; Pathak *et al.*, 2013). Absolute metabolite correction uses tissue water concentration to ‘water scale’ metabolite results and some studies enter the voxel fraction of CSF at this stage of analysis. This does not account for voxel differences in grey and white matter volume, which have different GABA and glutamate concentrations (Choi *et al.*, 2006; Gasparovic *et al.*, 2009; Bhattacharyya *et al.*, 2011). We used a generalized linear model, weighted for Cramér-Rao lower bound, to remove the effects of age, sex, grey and white matter from the results. This approach may still bias results if tissue volume closely correlates with metabolite concentration but, if anything, is likely to cause a type II error. Finally, nine patients were unable to complete the stop signal task, due to greater cognitive or motor impairment. This limits the applicability of these results to patients at the later stages of FTLD syndromes.

In conclusion, MRS has potential as an imaging biomarker of degeneration in bvFTD and PSP and possibly other syndromes associated with FTLD. In early bvFTD, there is selective vulnerability of glutamatergic von Economo neurons in the anterior cingulate and frontoinsular cortex (Seeley *et al.*, 2006; Kim *et al.*, 2012). MRS could enable *in vivo* quantification of this glutamatergic deficit, as an adjunct to studies of presymptomatic carriers of causative mutations (Rohrer *et al.*, 2015). Moreover, the association with neurotransmitter deficits and impaired response inhibition leads to the hypothesis that GABA reuptake inhibitors might be used to restore function (Adams *et al.*, 2020). Since behavioural disinhibition is associated with carer stress and poor patient outcome, symptom-oriented clinical trials are required for affected patients within the spectrum of disorders associated with FTLD.

## Supplementary Material

awaa305_Supplementary_Data

## Abbreviations

bvFTD =: behavioural variant frontotemporal dementia; FTLD = frontotemporal lobar degeneration; ^1^H-MRS = proton magnetic resonance spectroscopy; HDI = high density interval; PSP = progressive supranuclear palsy; SSRT = stop-signal reaction time
