# Supplementary material for: GABA and glutamate deficits from frontotemporal lobar degeneration are associated with disinhibition
Source: Brain. 2020 Nov 3;143(11):3449–62. doi: 10.1093/brain/awaa305 (PMC7719029; doi:10.1093/brain/awaa305)
Supplement: awaa305_Supplementary_Data [file awaa305_supplementary_data.pdf]

Supplementary Materials

Appendix 1: Stop/No-Go Task Details

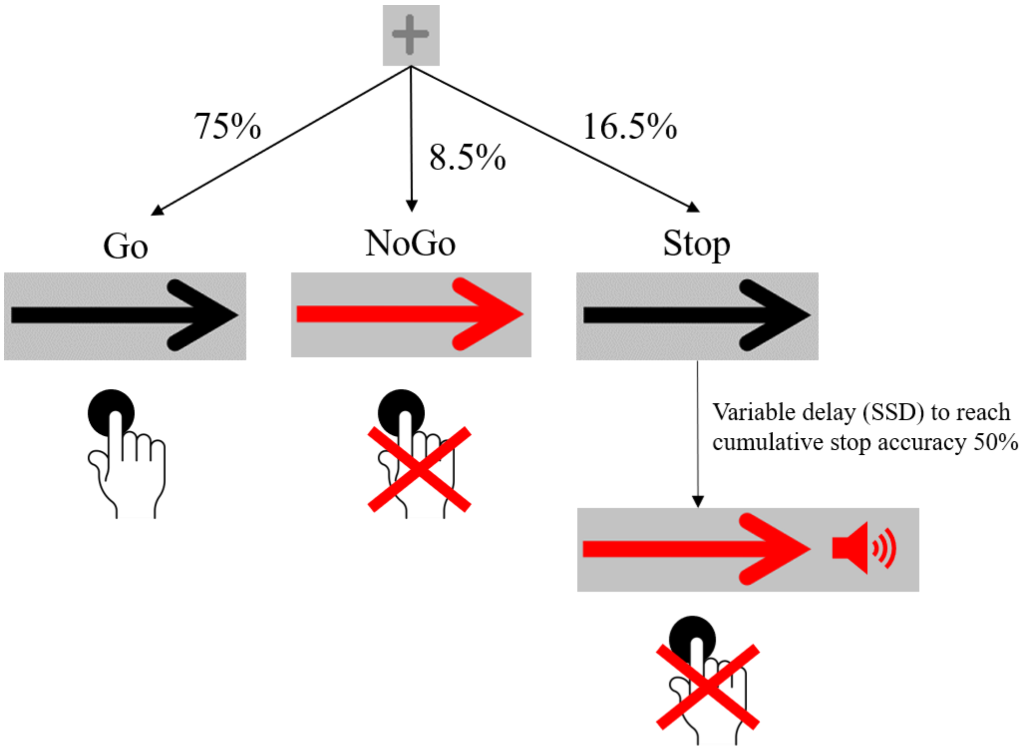

Figure S1: Description of the Stop No-Go task. Each trial started with a fixation cross, then either a go (black arrow) or no-go (red arrow) or stop (black arrow followed by red arrow and beep) stimulus presented. The delay between the go and stop arrow on stop trials (the stop signal delay) varied in a staircase algorithm to target a cumulative stop accuracy of 50%

Appendix 2: Partial volume correction of spectroscopy results

GABA and glutamate results were corrected for age, gender and tissue volume using a generalised linear model. The residuals from the generalised linear model $metabolite = \beta(intercept) + \beta(age) + \beta(gender) + \beta(grey\ matter)$ were used as the corrected results. A fourth covariate ($\beta(white\ matter)$) was added to the glutamate correction, as glutamate is present in both grey and white matter (Kukley *et al.*, 2007; Bakiri *et al.*, 2009). The absolute Cramer-Rao lower bound, a measure of LCModel fit accuracy, was used to weight the linear model (Miller *et al.*, 2017).

Appendix 3: Neuropsychology by FTLD syndrome subtype

Test	Control	bvFTD	PSP	F	p value	bvFTD vs Control		PSP vs Control		bvFTD vs PSP	
	mean (SD)					mean diff	p value	mean diff	p value	mean diff	p value
FTLD CDR SOB	0 (0)	12.86 (4.09)	7.32 (4.9)	61.95	2.05E-15	12.86	**2.05E-15**	7.32	**1.00E-07**	5.55	**2.11E-05**
PSPRS Total	0.1 (0.31)	13.95 (10.98)	31.45 (18.59)	32.38	2.61E-10	13.85	**2.61E-10**	31.35	**1.08E-09**	-17.5	**6.86E-05**
ACER Attention	17.95 (0.22)	12.18 (6.12)	16.23 (2.79)	11.95	4.19E-05	-5.77	**4.19E-05**	-1.72	3.41E-01	-4.05	3.41E-03
ACER Memory	21.3 (1.38)	12.14 (7.78)	19.59 (3.58)	19.69	2.52E-07	-9.16	**2.52E-07**	-1.71	5.25E-01	-7.45	**2.49E-05**
ACER Fluency	12.85 (1.04)	3.55 (3.42)	5 (2.94)	70.45	1.40E-16	-9.3	**1.40E-16**	-7.85	**9.57E-10**	-1.45	1.85E-01
ACER Language	25.5 (0.83)	16.77 (7.97)	23.27 (2.91)	17.54	9.58E-07	-8.73	**9.58E-07**	-2.23	3.26E-01	-6.5	**1.76E-04**
ACER Visuospatial	15.8 (0.52)	12.18 (5.21)	12.14 (3.81)	6.32	3.19E-03	-3.62	3.19E-03	-3.66	7.59E-03	0.05	9.99E-01
ACER Total	96.2 (2.71)	57.68 (28.1)	77.91 (13.17)	23.36	2.93E-08	-38.52	**2.93E-08**	-18.29	5.42E-03	-20.23	1.46E-03
FAB	17.45 (0.83)	9.55 (5.89)	12.55 (3.57)	20.06	2.01E-07	-7.9	**2.01E-07**	-4.9	6.88E-04	-3	4.49E-02
Hayling (A+B score)	4.3 (7.12)	33.15 (15.41)	15.76 (19.03)	20.05	2.03E-07	28.85	**2.03E-07**	11.46	4.06E-02	17.38	7.78E-04
Hayling Total	18.45 (2.28)	8.82 (3.94)	13 (5.14)	30.38	7.01E-10	-9.63	**7.01E-10**	-5.45	**1.27E-04**	-4.18	2.78E-03
INECO	25.78 (2.83)	10.44 (7.49)	17.45 (5.7)	37.39	2.51E-11	-15.34	**2.51E-11**	-8.33	**4.58E-05**	-7.01	**4.29E-04**

CBI Memory	2.06 (2.01)	18.27 (7.14)	7.18 (6.44)	44.08	1.43E-12	16.21	**1.43E-12**	5.12	1.49E-02	11.09	**7.61E-08**
CBI Everyday skills	0.15 (0.5)	10.68 (6.51)	8.64 (7.9)	17.81	8.10E-07	10.53	**8.10E-07**	8.48	**7.27E-05**	2.05	5.01E-01
CBI Selfcare	0.02 (0.09)	4.82 (4.44)	5.64 (6.2)	9.5	2.56E-04	4.8	**2.56E-04**	5.62	**4.08E-04**	-0.82	8.17E-01
CBI Behaviour	0.72 (0.93)	10.86 (6.79)	3.05 (3.18)	30.72	5.89E-10	10.14	**5.89E-10**	2.33	2.14E-01	7.82	**6.20E-07**
CBI Mood	0.86 (1.35)	4.64 (2.89)	2.55 (2.3)	14.29	8.15E-06	3.77	**8.15E-06**	1.68	5.34E-02	2.09	1.01E-02
CBI Abnormal beliefs	0 (0)	1.82 (2.13)	0.68 (1.21)	8.64	4.97E-04	1.82	**4.97E-04**	0.68	2.81E-01	1.14	2.93E-02
CBI Eating	0.3 (0.57)	9.18 (4.43)	3.68 (4.21)	32.78	2.15E-10	8.88	**2.15E-10**	3.38	9.66E-03	5.5	**1.18E-05**
CBI Sleep	0.89 (1.28)	3.77 (2.51)	3.18 (2.54)	9.81	2.02E-04	2.88	**2.02E-04**	2.29	3.85E-03	0.59	6.51E-01
CBI Motor behaviour	0.77 (1.21)	10.27 (4.99)	3.86 (4.79)	29.47	1.11E-09	9.51	**1.11E-09**	3.1	4.61E-02	6.41	**8.26E-06**
CBI Motivation/apathy	0.58 (0.94)	13.86 (4.7)	8.32 (6.78)	39.17	1.14E-11	13.28	**1.14E-11**	7.74	**8.93E-06**	5.55	1.04E-03
CBI Impulsivity*	2.32 (2.73)	33.91 (16.21)	11.82 (10.12)	43.63	1.73E-12	31.59	**1.73E-12**	9.5	2.30E-02	22.09	**5.56E-08**
CBI Total	6.35 (6.13)	88.18 (31)	46.77 (33.24)	48.58	2.40E-13	81.83	**2.40E-13**	40.42	**2.48E-05**	41.41	**1.02E-05**
FRS Total (Logit)	0.86 (0.3)	0.23 (0.16)	0.48 (0.31)	29.3	1.21E-09	-0.62	**1.21E-09**	-0.37	**7.25E-05**	-0.25	7.34E-03

Neuropsychological tests: Neuropsychology of FTLD syndromes (bvFTD and PSP). CDR-FTLD SOB: Clinical Dementia Rating scaling sum of boxes modified for FTLD. PSPRS: Progressive Supranuclear Palsy rating scale. ACER: Addenbrooke's Cognitive Examination-Revised. FAB: Frontal Assessment Battery. CBIR: Cambridge Behavioural Inventory Revised. FRS: Frontotemporal Dementia Rating Scale. *CBI Impulsivity score calculated from all items from the disinhibited, challenging, motor, eating and insight subscales and the euphoria items from the mood subscale (Borroni *et al*, 2012). P values in bold remain significant ($p<0.05$) after Bonferroni correction ($6.6x10^{-04}$).

Appendix 4: Voxel based morphometry

VBM Methods

Voxel-based morphometry was performed with the standard settings in SPM12 (Ashburner and Friston, 2000; Ashburner and Reg, 2010). First, MP2RAGE images were aligned to an average image in MNI space, cropped to a standard bounding box then segmented into six tissue probability maps: grey matter, white matter, CSF, bone, soft tissue and air. A study-specific template was created using diffeomorphic anatomical registration using exponentiated Lie algebra (DARTEL) on images from all participants. The tissue probability maps for each participant were then warped to this template. Next, the grey and white matter templates were affine transformed and warped to MNI space. This transformation was then applied to each participant's tissue probability images. Smoothing was performed with an 8mm isotropic full width at half maximum Gaussian kernel. The total intracranial volume for each participant was calculated using the Tissue Volumes function in SPM12. Study-specific grey and white matter masks were created from voxels with a value of >0.15 in more than half of the images (Ridgway *et al.*, 2009). An average of the all participants' structural images was created to visualise thresholded cluster maps and spectroscopy voxels. After skull stripping, all images were normalised using the study specific DARTEL template, but with no smoothing or modulation. An averaged image was created using the *AverageImages* function in ANTS (Avants *et al.*, 2009).

Grey and white matter volumes for each diagnostic group were compared with independent two-sample t-tests with age, sex and total intracranial volume as covariates of no interest (Barnes *et al.*, 2010). The conjunction between bvFTD and PSP was tested on the combined pairwise contrasts (bvFTD vs control and PSP vs control) on an ANCOVA across all groups with the same covariates of no interest (Nichols *et al.*, 2005). Significant effects were identified using cluster-level statistics ($p<0.05$, family-wise error corrected for multiple comparisons) above a height threshold of $p<0.001$ (uncorrected).

VBM Results

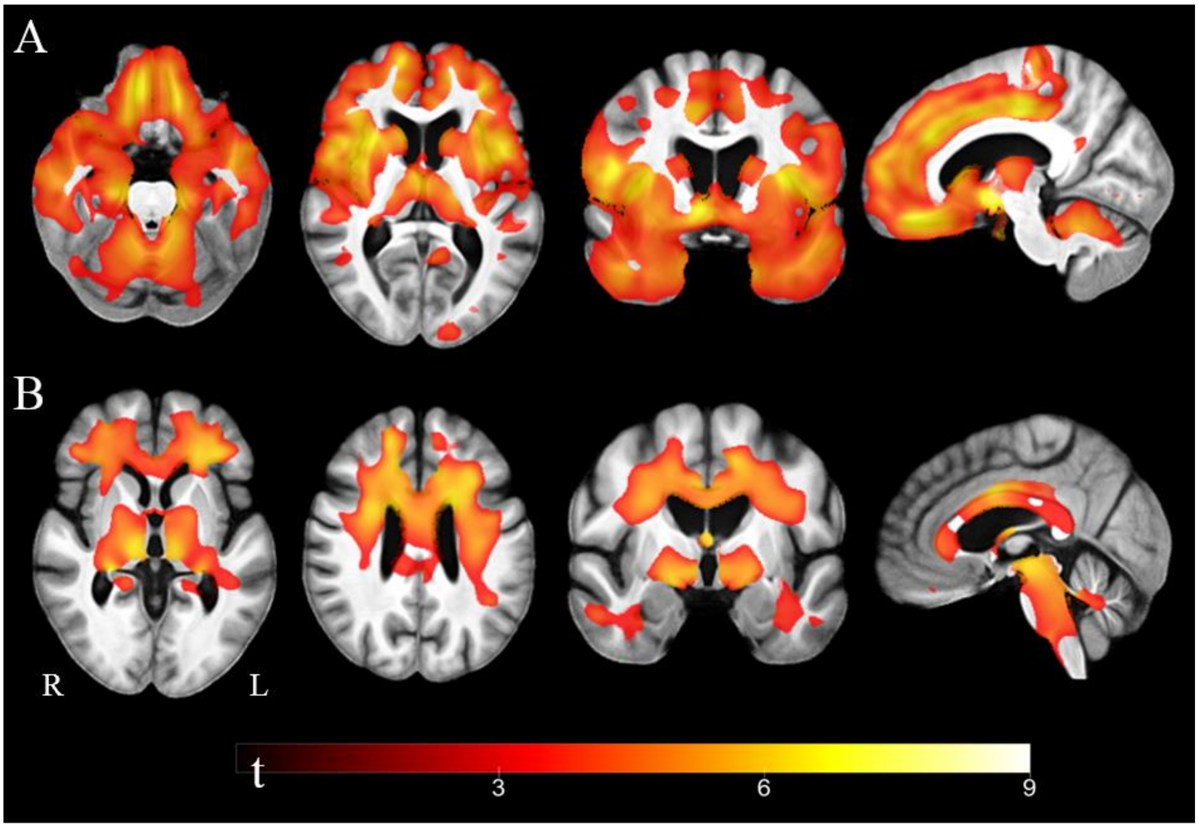

Figure S2: Voxel based brain morphometry of FTLD (bvFTD and PSP combined). A: Grey matter B: White matter. Representative axial, coronal and sagittal slices are shown.

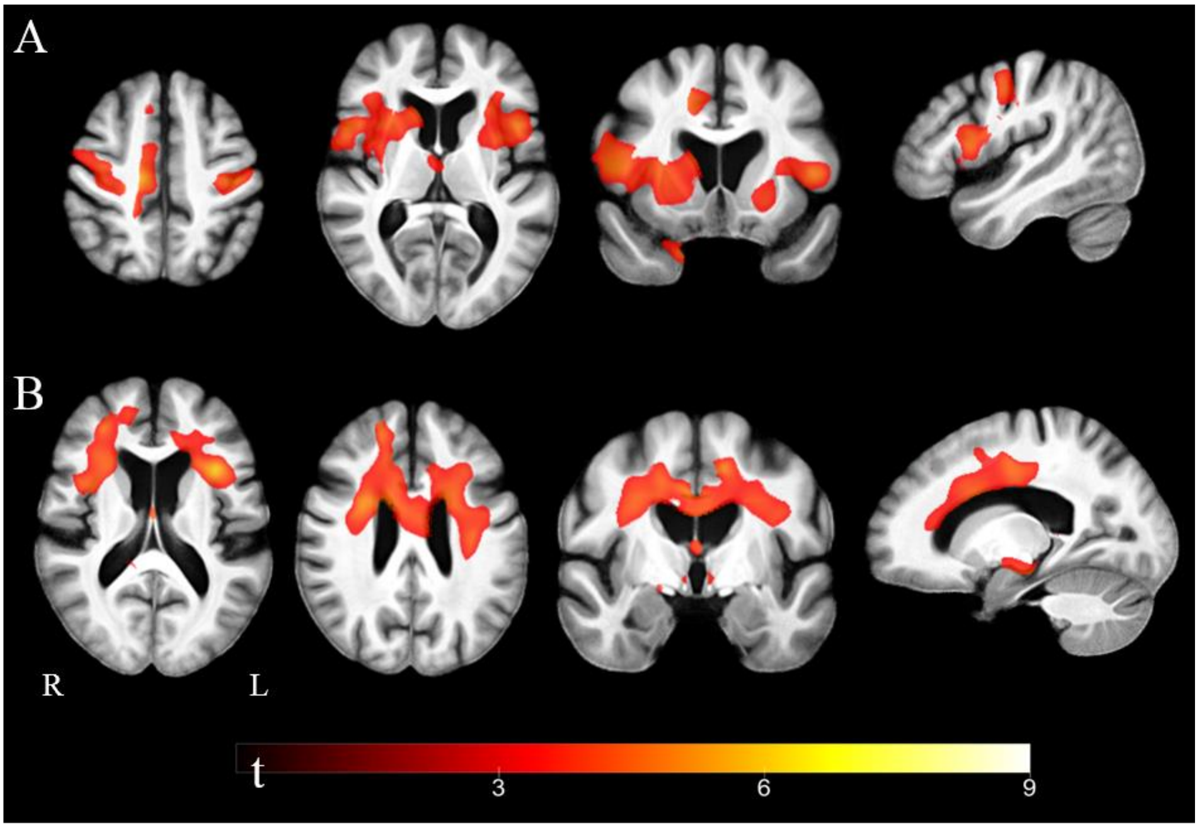

Figure S3: Conjunction null analysis of bvFTD vs Control and PSP vs Control. The colourmap shows voxels that are significant in both groups, at a cluster-level of FWE $p<0.05$ above a height threshold of $p<0.001$. A: Grey matter. B: White matter. Representative axial, coronal and sagittal slices are shown.

Appendix 5: Water scaled MRS results without partial volume correction

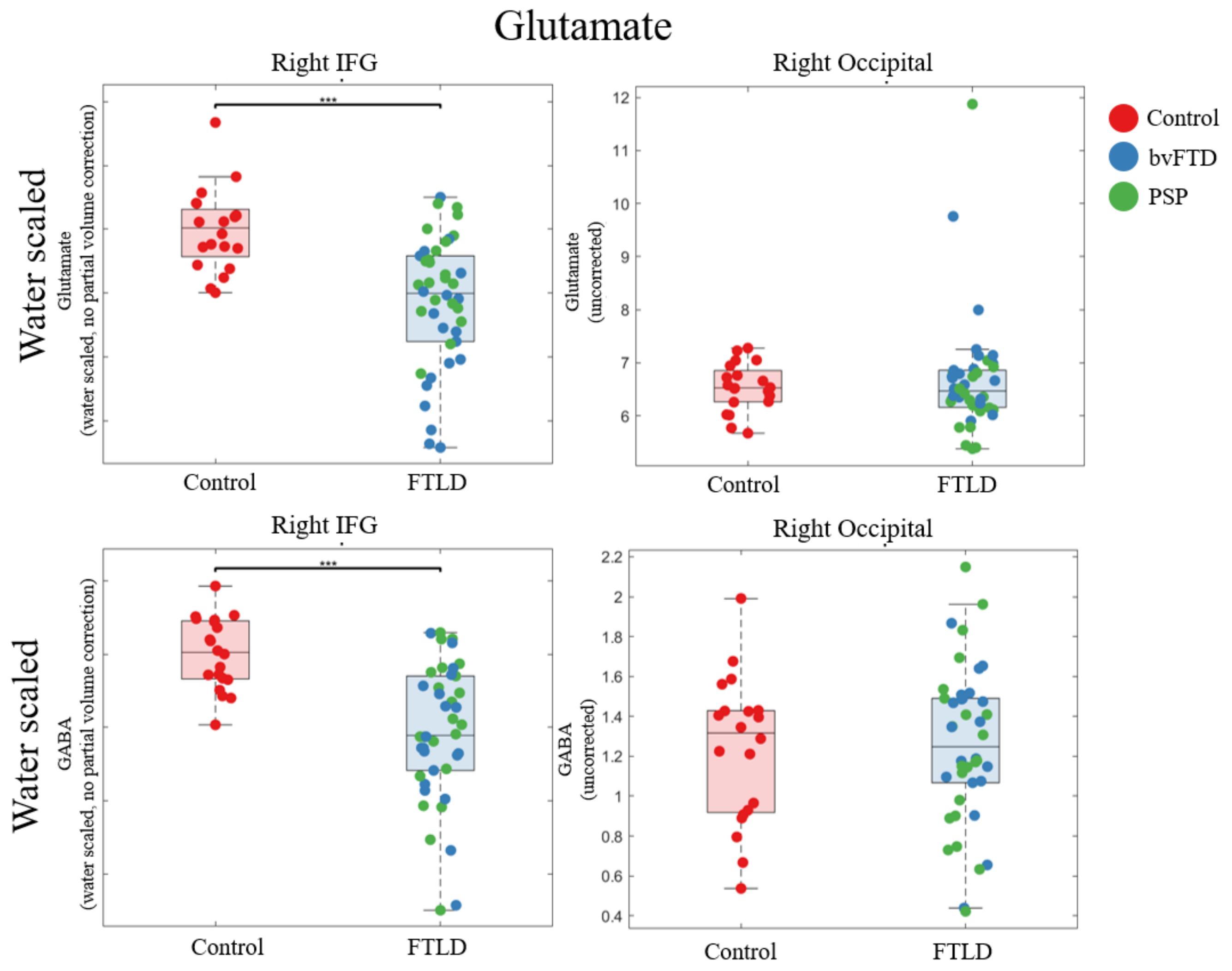

Figure S4. Uncorrected Glutamate and GABA concentrations in the FTLD syndromes of bvFTD and PSP. GABA and glutamate values were water scaled using an unsuppressed water peak acquired as part of the spectroscopy sequences, but not corrected for the fraction of brain volume in the MRS voxel. ***:p<0.001. FTLD: Frontotemporal lobar degeneration syndrome, bvFTD/PSP-RS/PSP-F. IFG: Inferior frontal gyrus.

Appendix 6: Stop No-Go Task Results

	Control	FTLD (bvFTD+PSP)	bvFTD	PSP
**Total trials (n)**	670.05 (92.36)	663.14 (97.91)	636.47 (122.34)	687 (63.76)
**Go correct (n)**	520.7 (68.56)	489.00 (81.90)	467.77 (99.91)	508 (57.99)
**Go incorrect (n)**	6.8 (6.13)	29.28 (42.16)	27.88 (53.34)	30.53 (30.4)
**Go Omission (n)**	0.05 (0.22)	4.42 (15.52)	7.53 (22.31)	1.63 (2.99)
**NoGo Correct (n)**	45.4 (7.98)	42.11 (15.61)	37.53 (15.24)	46.21 (15.17)
**NoGo incorrect (n)**	2.1 (3.6)	6.08 (9.06)	6.88 (10.21)	5.37 (8.12)
**Stop correct (n)**	41.45 (8.78)	30.64 (10.43)	31.47 (12.02)	29.9 (9.04)
**Stop failed/incorrect (n)**	53.55 (8.19)	61.61 (11.66)	57.41 (11.95)	65.37 (10.29)
**Go correct reaction time (ms)**	641.77 (124.3)	1082.64 (344.61)	1023.6 (335.87)	1135.46 (352.71)
**Go error rate**	0.01 (0.01)	0.06 (0.09)	0.07 (0.12)	0.06 (0.06)
**NoGo error rate**	0.04 (0.07)	0.14 (0.22)	0.18 (0.27)	0.11 (0.16)
**Stop accuracy rate**	0.43 (0.04)	0.33 (0.09)	0.35 (0.1)	0.31 (0.08)

Table of stop no-go behavioural results. Each cell contains mean and (standard deviation).

Appendix 7: Dynamic Models of Choice fitting

MCMC chains – Control model

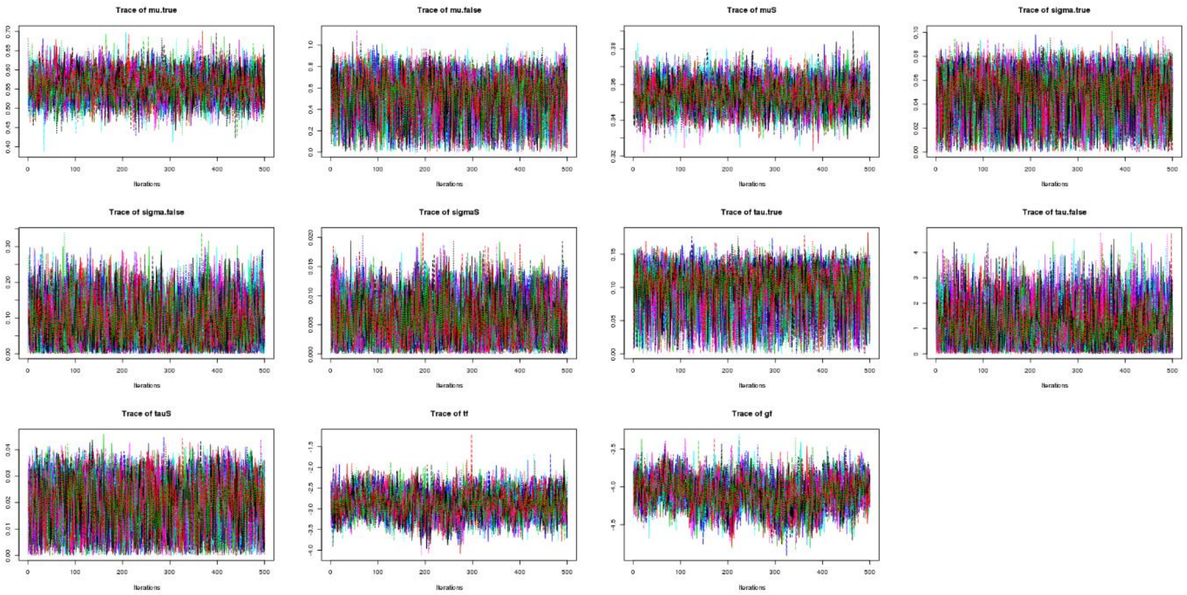

MCMC chains – FTLD model

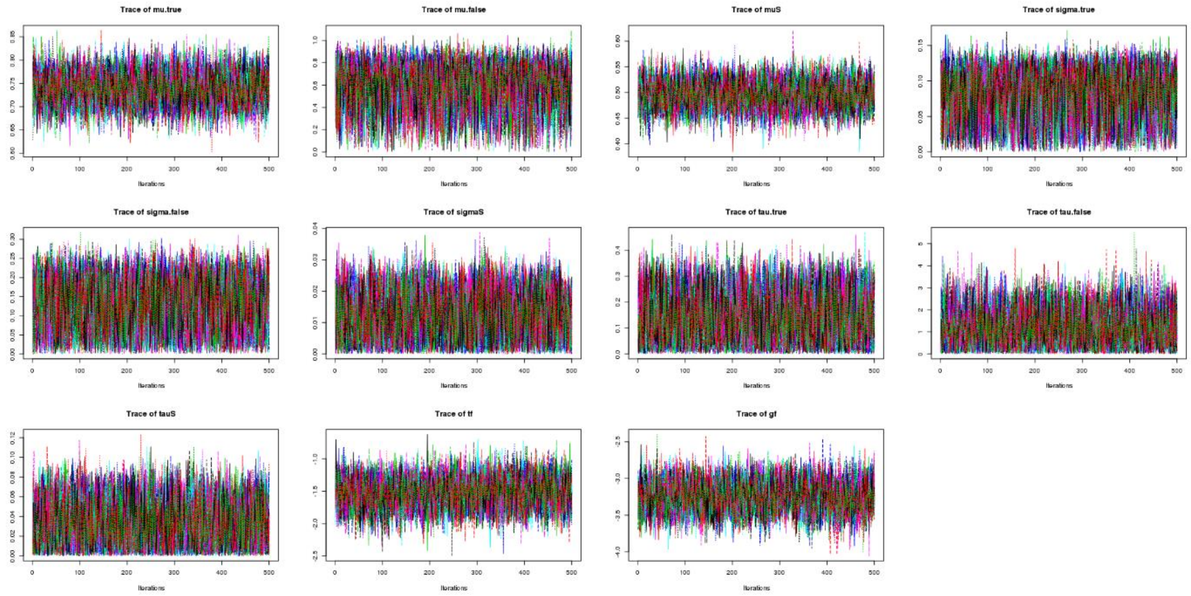

Figure S5: Plots of Markov chain Monte Carlo (MCMC) chains comprising the final 500 iterations for each estimated parameter.

Prior and posterior density plots

Controls

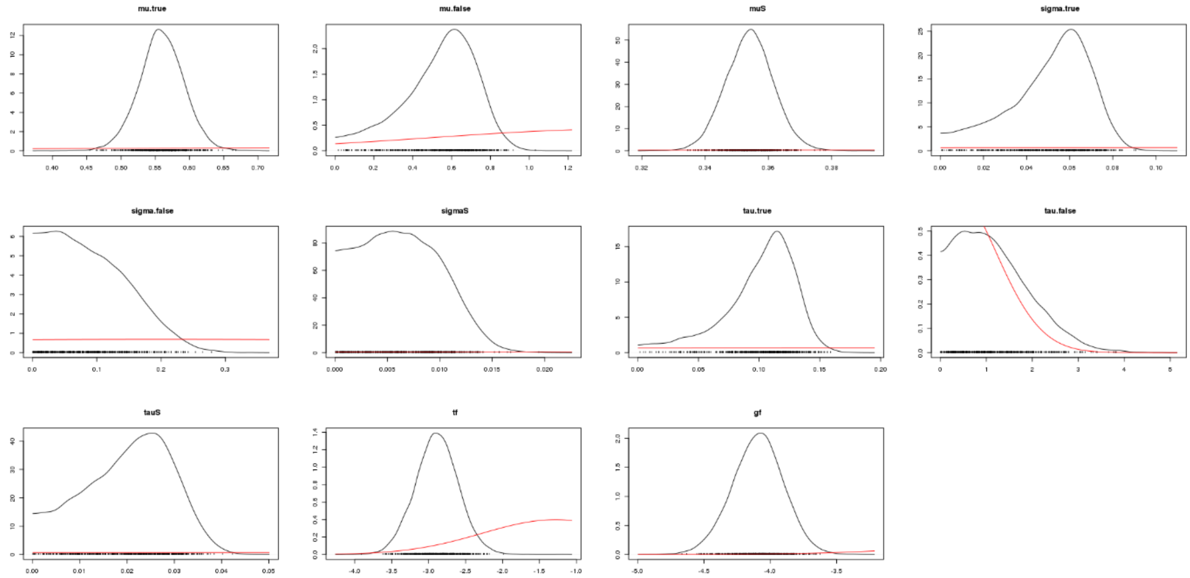

FTLD

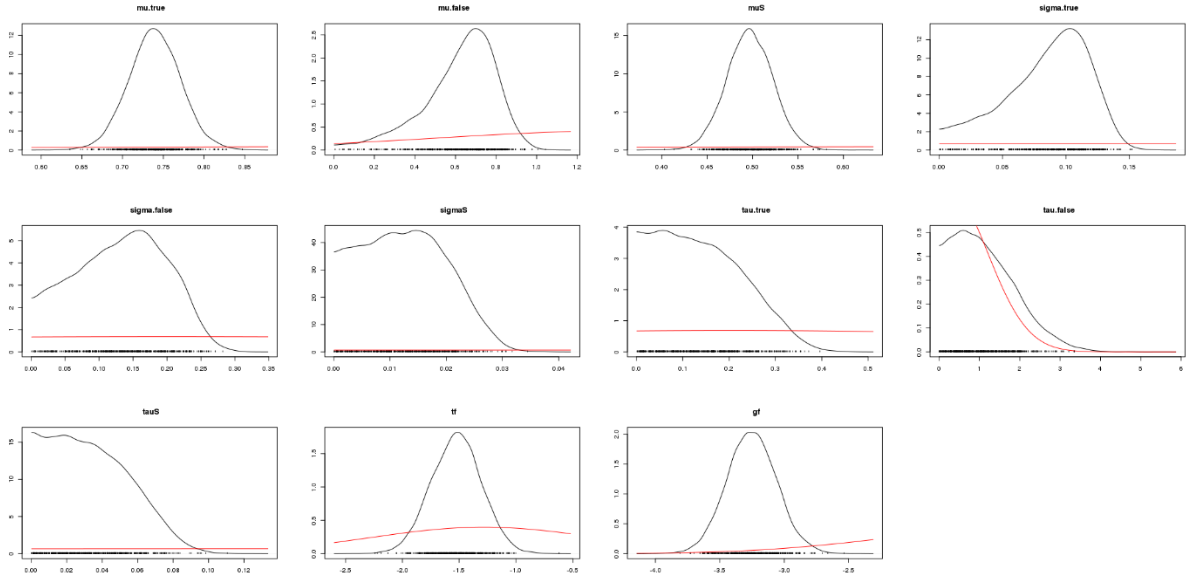

Figure S6. Hyper-prior (red lines) and posterior (black peaked lines) distributions for the population means.

Goodness of fit results

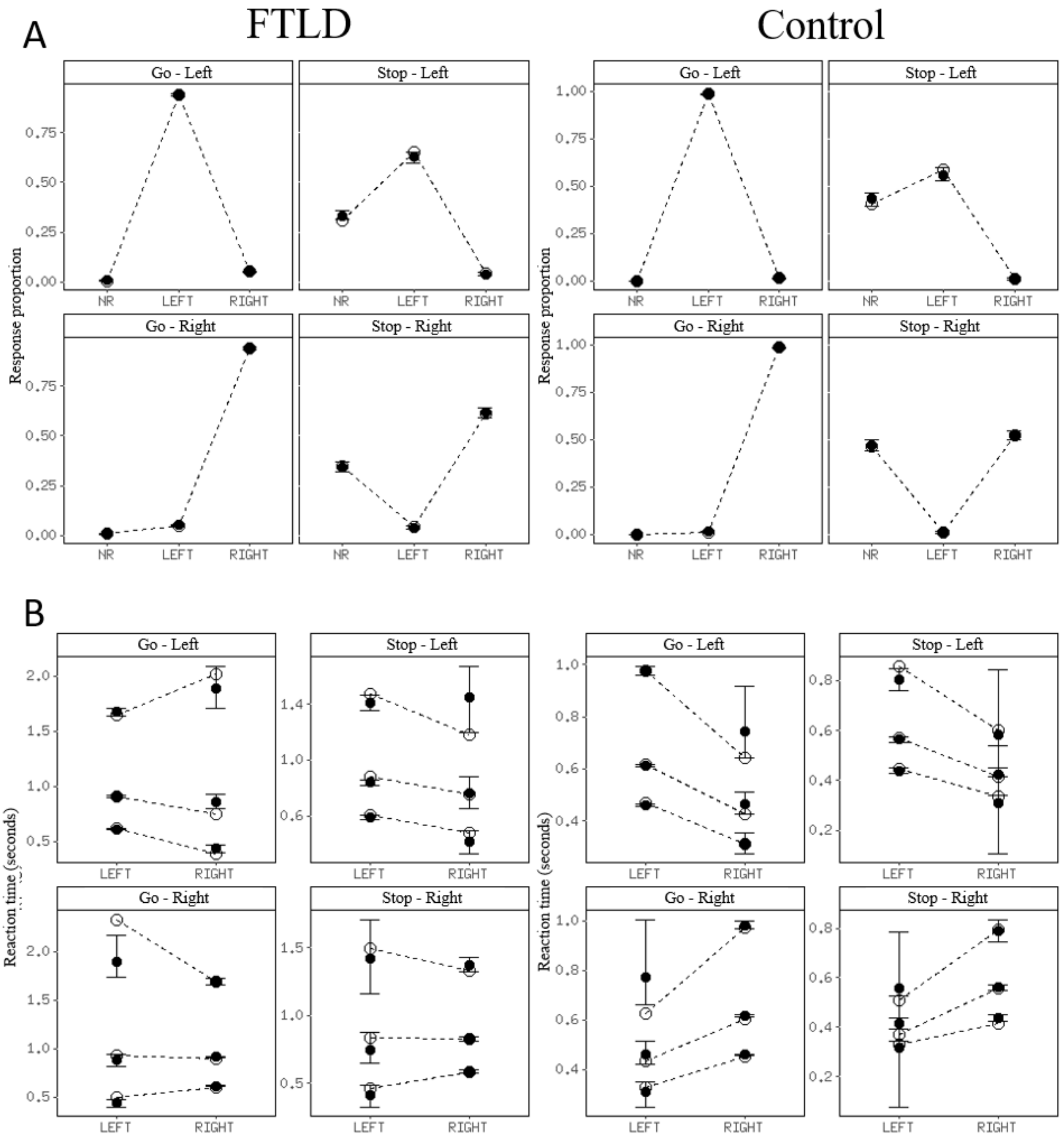

Figure S7. Goodness of fit functions test if the model accurately represents the data by comparing experimentally acquired data to simulated results from the final model fit. A: Response proportions (NR=non-response). B: Response time percentiles. The three lines correspond to the 10th, 50th, and 90th percentiles. Dashed line and open points represent the data. Solid points represent medians of the model prediction. Error bars show the 95% credible intervals. The figures show average results over all participants.

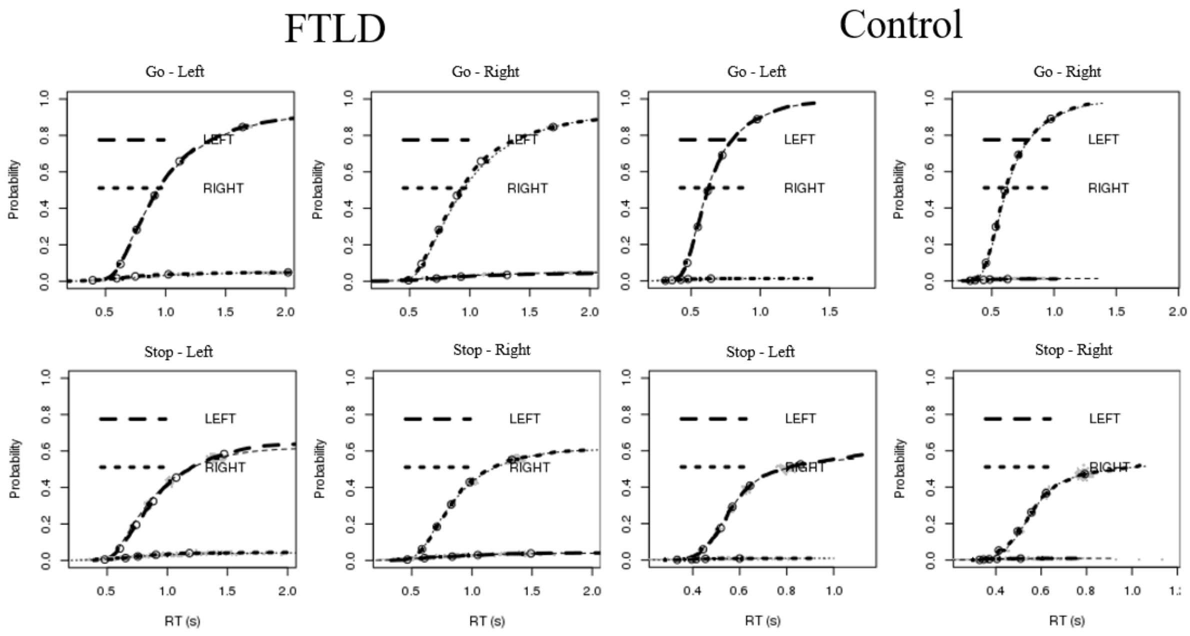

Figure S8. Cumulative distribution probability functions. Thick lines represent the data, thin lines represent model predictions. Open points mark the 10th, 30th, 50th, 70th, and 90th percentiles. The clusters of grey dots represent the uncertainty in the percentiles from 100 randomly selected samples from the joint posterior

Appendix 8: Comparison of cognitive tests and metabolites

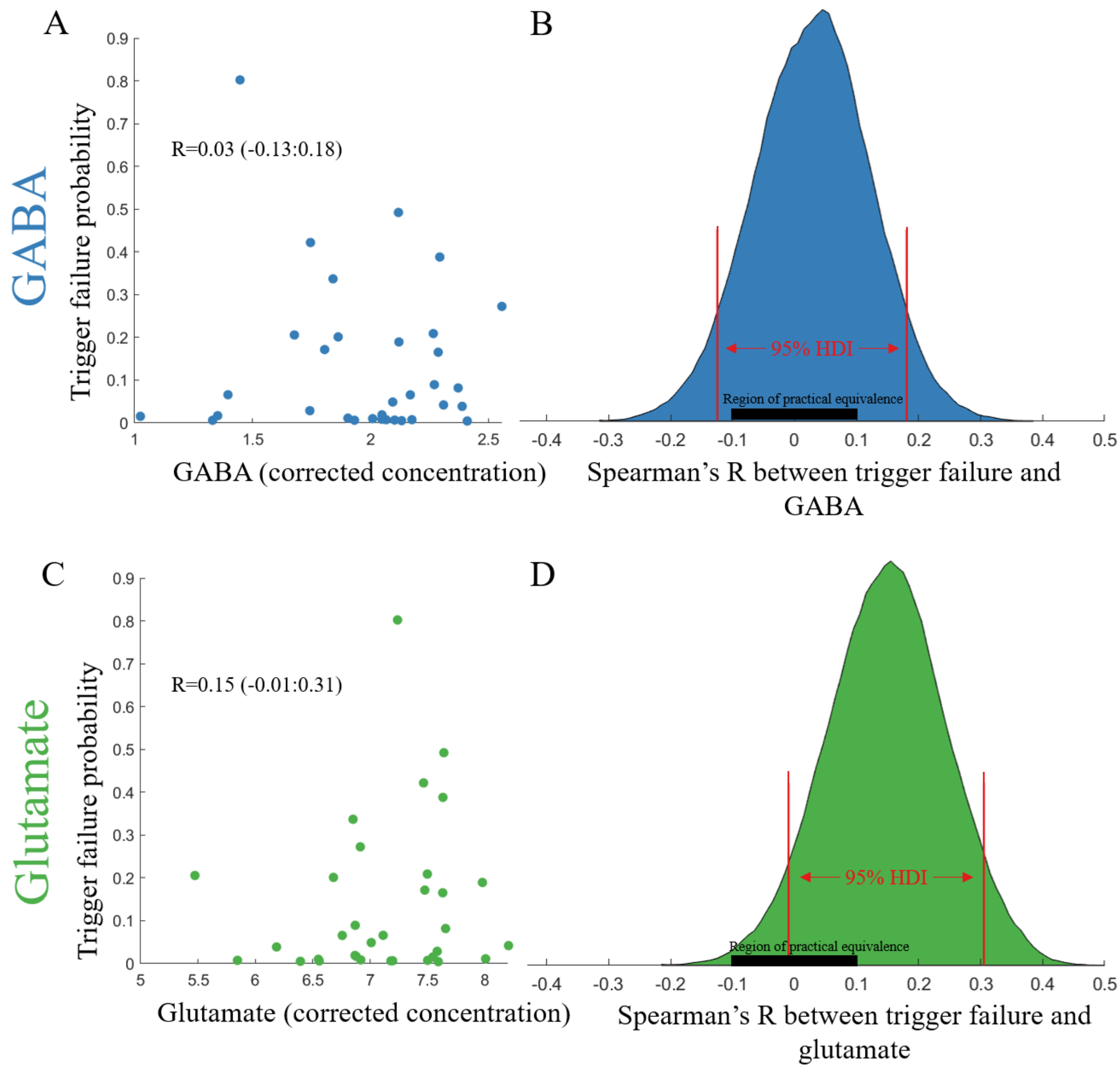

Figure S9. Correlation between neurotransmitters (GABA and glutamate) and trigger failure probability. Results from bvFTD/PSP patients only. A: Scatter plot of median trigger failure probability and corrected GABA, values in brackets are 95% HDI. B: Histogram of Spearman's correlation values between glutamate (corrected for grey matter, age and sex) and trigger failure probability. Red lines show 95% highest density interval (HDI). Black bar shows region of practical equivalence (-0.1, 0.1). C: Histogram of Spearman's correlation values between glutamate and trigger failure probability. D: Scatter plot of median trigger failure probability and corrected glutamate

	CDR-SOB	ACER Attention	ACER Memory	ACER Fluency	ACER Language	ACER Visuospatial	ACER Total	FAB	Hayling A+B Score	INECO	CBI Impulsivity	CBI Total	FRS Total
NAA	-0.24 (0.12)	0.18 (0.24)	0.25 (0.11)	0.31 (0.04)	0.14 (0.37)	0.07 (0.67)	0.22 (0.16)	0.08 (0.62)	-0.24 (0.12)	0.16 (0.31)	-0.11 (0.49)	-0.15 (0.35)	0.21 (0.18)
Cr/PCr	-0.12 (0.43)	0.1 (0.53)	0.18 (0.24)	0.12 (0.45)	0.11 (0.49)	-0.11 (0.5)	0.11 (0.49)	-0.03 (0.84)	-0.34 (0.03)	0.09 (0.56)	-0.04 (0.79)	-0.02 (0.9)	0.04 (0.81)
GPC/PCh	-0.14 (0.37)	0.18 (0.24)	0.19 (0.23)	0.16 (0.3)	0.16 (0.3)	-0.03 (0.82)	0.17 (0.29)	0.06 (0.72)	-0.03 (0.87)	0.12 (0.43)	-0.08 (0.59)	-0.1 (0.53)	0.02 (0.9)
Myo-inositol	0.16 (0.32)	-0.1 (0.52)	-0.09 (0.56)	-0.09 (0.57)	-0.08 (0.6)	-0.02 (0.88)	-0.08 (0.6)	-0.07 (0.64)	0.04 (0.82)	-0.06 (0.71)	0.27 (0.08)	0.18 (0.25)	-0.06 (0.72)
Glutamine	-0.27 (0.08)	0.08 (0.59)	0.01 (0.96)	-0.1 (0.53)	-0.02 (0.89)	-0.01 (0.94)	0 (0.98)	0.14 (0.37)	-0.1 (0.54)	0.21 (0.17)	-0.16 (0.31)	-0.19 (0.22)	0.22 (0.16)
Glutathione	-0.01 (0.96)	0.1 (0.53)	0.12 (0.44)	0.07 (0.63)	0.06 (0.69)	0.12 (0.45)	0.11 (0.47)	0.01 (0.94)	0.07 (0.64)	0.03 (0.87)	0.18 (0.25)	0.12 (0.43)	-0.06 (0.72)
GABA	-0.27 (0.08)	0.26 (0.1)	0.18 (0.25)	0.11 (0.48)	0.17 (0.26)	0.31 (0.04)	0.23 (0.13)	0.21 (0.17)	-0.02 (0.88)	0.15 (0.32)	-0.03 (0.84)	-0.12 (0.46)	0.31 (0.04)
Glutamate	-0.15 (0.35)	0.14 (0.37)	0.17 (0.28)	0.33 (0.03)	0.02 (0.9)	0.01 (0.93)	0.14 (0.36)	0.22 (0.16)	-0.15 (0.34)	0.23 (0.13)	0.07 (0.65)	-0.01 (0.97)	0.13 (0.41)

Table of correlations between neuropsychological tests and carer rating scales and other MRS-visible metabolites. Metabolites corrected as per Appendix 2. Values shown are Pearson's R and (p values). None of the correlations were significant after correction for multiple comparisons (Bonferroni p threshold: 0.00048). Cr/PCr: creatine/phosphocreatine, GPC/PCh: Glycerophosphocholine/phosphocholine, NAA: N-acetylaspartate, CDR-SOB: Clinical Dementia Rating Scale FTLD version Sum of Boxes, ACER: Addenbrooke's Cognitive Examination – Revised, FAB: Frontal Assessment Battery, Hayling: Hayling Inhibition Score, INECO Frontal Assessment Battery, CBI: Cambridge Behavioural Inventory, FRS: Frontotemporal Dementia Rating Scale.

References

Ashburner J, Friston KJ. Voxel-based morphometry - The methods. Neuroimage 2000; 11: 805–821.

Ashburner J, Reg C. VBM tutorial_ja. Options 2010: 1–18.

Avants BB, Tustison N, Song G. Advanced normalization tools (ANTS). Insight j 2009; 2: 1–35.

Barnes J, Ridgway GR, Bartlett J, Henley SMD, Lehmann M, Hobbs N, et al. Head size, age and gender adjustment in MRI studies: A necessary nuisance? Neuroimage 2010; 53: 1244–1255.

Borroni B, Grassi M, Premi E, Gazzina S, Alberici A, Cosseddu M, et al. Neuroanatomical correlates of behavioural phenotypes in behavioural variant of frontotemporal dementia. Behav Brain Res 2012; 235: 124–129.

Nichols T, Brett M, Andersson J, Wager T, Poline JB. Valid conjunction inference with the minimum statistic. Neuroimage 2005; 25: 653–660.

Ridgway GR, Omar R, Ourselin S, Hill DLG, Warren JD, Fox NC. Issues with threshold masking in voxel-based morphometry of atrophied brains. Neuroimage 2009; 44: 99–111.
